# Silicon Confers Salt Tolerance in Potato in a Concentration-Dependent Manner via Coordinated Antioxidant and Osmotic Regulation

**DOI:** 10.3390/antiox15101247

**Published:** 2026-09-27

**Authors:** Panfeng Yao, Junmei Cui, Wenlin Li, Ying Wang, Jianguo Wei, Zhenzhen Bi, Chao Sun, Zhen Liu, Han Wang, Zhanshu Song, Yuhui Liu, Jiangping Bai

**Affiliations:** 1 State Key Laboratory of Aridland Crop Science, Gansu Agricultural University, Lanzhou 730070, China; yaopf@gsau.edu.cn (P.Y.);; 2 College of Agronomy/Seed Industry Research, Institute of Gansu Provincial University, Gansu Agricultural University, Lanzhou 730070, China; 3 College of Horticulture, Gansu Agricultural University, Lanzhou 730070, China; 4 Qingyang City Academy of Agricultural Sciences, Qingyang 745000, China; 5 Lanzhou Institute of Technology, Lanzhou 730070, China

**Keywords:** potato, salt stress, Si, WGCNA, antioxidant enzymes

## Abstract

Soil salinization restricts potato yield by inducing excessive reactive oxygen species (ROS) accumulation and oxidative damage. Exogenous silicon (Si) can alleviate salt-induced oxidative injury, yet the concentration-dependent mechanisms remain unclear in potato. Here, we conducted a tiered investigation combining phenotypic, physiological, and transcriptomic analyses. An in vitro gradient screening under 0.3% NaCl stress identified 1.0 mM Si as optimal. Compared with NaCl-alone treatment, 1.0 mM Si enhanced Catalase (CAT), Peroxidase (POD), and Superoxide Dismutase (SOD) activities by approximately 2.1-fold, 2.3-fold, and 1.3-fold, respectively. This Si supplementation also dramatically reduced ROS accumulation and membrane lipid peroxidation, as evidenced by 3,3′-diaminobenzidine (DAB) and nitroblue tetrazolium (NBT) staining and decreased Malondialdehyde (MDA) content. Subsequent pot experiments under 100 and 200 mM NaCl confirmed that 2.0 mM Si most effectively alleviated oxidative injury, accompanied by increased proline, higher relative water content, and reduced ROS and MDA. These data demonstrated that Si exerts dose-dependent protection by orchestrating both enzymatic (SOD, POD, CAT) and non-enzymatic (proline, glutathione) antioxidant systems. Transcriptome profiling coupled with Weighted Gene Co-expression Network Analysis (WGCNA) and RT-qPCR revealed eight candidate hub genes, with antioxidant defense-related *StGSH1* (glutathione synthesis) and *StPRX52* (peroxidase) as key components, alongside genes involved in protein homeostasis and osmotic adjustment. Collectively, our findings establish optimal Si regimes and demonstrate that Si mitigates salt injury primarily through enhancing antioxidative capacity and ROS detoxification, providing candidate genes for antioxidative breeding in potato.

## 1. Introduction

Potato is an important staple crop globally, ranking fourth in the world in terms of production, after rice, wheat, and corn [1]. Potato is highly sensitive to salt stress, a major constraint on its cultivation [2]. Severe stress can cause a drastic reduction in tuber yield [3,4]. Salt stress induces metabolic disturbances, water dysregulation, and ROS-mediated cellular injury in potato, impairing stolon initiation and vegetative development [5,6]. Because the early growth stage is particularly vulnerable, salt toxicity reduces leaf number, leaf water potential, chlorophyll content, dry matter accumulation, and root/stem K^+^ concentrations, ultimately hindering tuber yield [7,8]. Therefore, improving salt tolerance and breeding salt-tolerant varieties in potato has become an urgent priority. However, the molecular basis of salt tolerance in potato remains poorly understood, and the identification of functional genes and regulatory networks underlying stress responses is urgently needed.

Salinity stress is one of the most severe abiotic stress factors that constrain plant growth, development, and productivity [9]. Approximately 20% of the world’s cultivated land and 33% of its irrigated farmland are threatened by high salinity, and the area of salinized land is expanding year by year [10]. Worldwide, about 1.0 × 10^9^ hm^2^ of land is subject to salinization, posing a significant constraint on agricultural productivity [11]. Studies have shown that salinity stress can lead to a yield loss of up to 40% in crops, and the occurrence of such stress is closely related to various environmental factors, with sodium chloride (NaCl) being one of the major salt constituents responsible for it [12]. Under salinity stress, plants accumulate stress markers such as reactive oxygen species (ROS) and malondialdehyde (MDA). These substances attack the cell membrane system via oxidative stress, triggering membrane lipid peroxidation, abnormal membrane permeability, and organelle dysfunction, which subsequently lead to cell death [13]. Ultimately, this cascade of events systematically impairs vegetative growth, reproductive development, and the overall metabolic network of plants [14]. Prolonged salinity stress gradually reduces stomatal conductance and limits intercellular CO_2_ supply, thereby suppressing net photosynthetic rate and transpiration rate [15]. If the stress persists, degradation of photosynthetic pigments may also occur [16]. Elevated salt ion concentrations lower the osmotic potential of the growth medium, thereby inducing physiological drought in plants [17]. NaCl serves as the major contributor to salt stress, and the excessive Na^+^ it liberates competes with K^+^ for root absorption sites, resulting in overaccumulation of Na^+^ and disruption of Na^+^/K^+^ homeostasis, which ultimately impairs plant growth and metabolism [18].

Silicon (Si) is the second most abundant element in the Earth’s crust. Therefore, plants growing in soil generally accumulate detectable levels of Si in their tissues [19]. Silicon has been demonstrated to substantially improve salt tolerance in multiple crops, such as soybean [20], maize [21], and rice [22], through mechanisms involving enhanced water retention, increased cell-wall rigidity, improved stem-lodging resistance, and elevated chlorophyll levels and photosynthesis [23]. Silicon alleviates salt stress by reducing sodium absorption and its translocation to the aboveground parts. This effect is partly attributed to Si-driven physical modifications in root tissues: Si deposition within root cell-wall matrices may reinforce apoplastic barriers and restrict the apoplastic bypass flow of Na^+^ toward vascular tissues, although such direct mechanisms remain poorly characterized in potato. Nevertheless, the bioavailability of silicon in soil is typically limited, which has historically restricted most studies to a narrow range of Si-accumulating crops [24]. Silicon supplementation has been reported to exert alleviating effects under various stresses, including salinity [25], high temperature [26], powdery mildew [27], waterlogging [28], and herbivory [29]. In addition, silicon can enhance crop yield and disease resistance, and also shows general benefits in mitigating abiotic stresses [30]. As an emerging green agronomic practice, exogenous silicon treatment shows promising application prospects in alleviating salt stress in plants, since silicon is a naturally-occurring mineral element without toxic residues, and its application does not bring risks of environmental pollution compared with chemical regulators [31]. Although the beneficial effects of silicon on plant growth under stress have been well documented, the underlying mechanisms remain elusive and warrant further investigation [32].

Reactive oxygen species (ROS) are a class of multifunctional signaling molecules that are induced by various environmental stresses, including salinity. However, over accumulated ROS is toxic to plants, leading to structural disruption of cells and promoting accelerated cell death [33]. Previous studies have shown that silicon application under 120 mM NaCl stress significantly enhanced the activities of major antioxidant enzymes and increased the content of the non-enzymatic antioxidant glutathione (GSH) in barley. Exogenous silicon significantly improved salt tolerance in barley by strengthening the root antioxidant defense system and alleviating lipid peroxidation [34,35]. Plants typically maintain ROS homeostasis via an antioxidant defense system comprising enzymatic antioxidants (SOD, POD, CAT) and non-enzymatic antioxidants (ascorbic acid and glutathione). Silicon application under salt stress is known to lower ROS accumulation and improve membrane lipid stability, though its effectiveness is species dependent. Silicon elevated SOD activity but not CAT activity in salt-treated cucumber leaves, and further noted that such modulation is influenced by cultivar and stress duration [36].

Considering the incomplete understanding of silicon-driven salt-tolerance mechanisms in potato, the present study adopted a tiered strategy combining physiological phenotyping and transcriptomic dissection. We screened optimal Si dosage via in vitro assays, validated the concentration-dependent amelioration under mild and severe salt stress in pots, and profiled root and shoot transcriptomes to mine hub genes through WGCNA and RT-qPCR verification. We aimed to decipher how Si coordinates redox, osmotic, and protein homeostasis to mitigate salt injury. These findings will support Si fertilizer utilization and provide candidate genes for potato salt-tolerance improvement.

## 2. Materials and Methods

### 2.1. Plant Materials

#### 2.1.1. Tissue Culture Experiment

Virus-free tissue-cultured plantlets of the potato cultivar ‘Atlantic’ were provided by Gansu Agricultural University. Uniform and healthy plantlets were inoculated onto MS media with different treatments. Five treatment groups were established: normal MS medium (CK, control), MS medium supplemented with 0.3% NaCl (salt stress), and MS medium containing 0.3% NaCl plus 0.5 mM Si (A1), 1.0 mM Si (A2), 2.0 mM Si (A3), or 3.0 mM Si (A4).

Silicon was added directly into MS medium using sodium silicate nonahydrate (Na_2_SiO_3_·9H_2_O), and all Si concentrations were calculated on an elemental-Si basis. For this in vitro experiment, each treatment contained three independent biological replicates, and each replicate consisted of 12 individual plantlets. Physiological phenotype observations, histochemical DAB and NBT staining, antioxidant-enzyme activity measurements, related biochemical assays, and transcriptome sampling for RNA-seq were performed exclusively using these tissue-cultured plantlets. Phenotypic evaluation and sample collection were conducted at 28 days post-inoculation, after 28 days of continuous stress treatment. After inoculation, all cultures were placed in a growth room and maintained under closed conditions for 28 days. The environmental conditions were set as follows: photoperiod of 16 h light/8 h dark, photosynthetic photon flux density of 250 μmol photons m^−2^ s^−1^, temperature of 22 ± 2 °C, and relative humidity of 60%.

#### 2.1.2. Pot Experiment

Potatoes of the cultivar ‘Atlantic’ were planted in plastic pots (13 cm × 13 cm) filled with a substrate consisting of soil and vermiculite at a volume ratio of 2:1, with one plant per pot. Seed-tuber-derived seedlings were grown for approximately 40 days under normal conditions until they developed 8–10 true leaves with plant height reaching 15–20 cm; plants with uniform growth status were then selected to initiate salt and exogenous silicon stress treatments. Two salt concentration levels were established: 100 mM NaCl and 200 mM NaCl. From the start of treatment, the corresponding NaCl solution was irrigated every 3 days for a total of five consecutive applications. Exogenous silicon treatment was initiated on the 3rd day after the first salt application. Under each salt concentration, silicon solutions at 1 mM (S1), 2 mM (S2), and 3 mM (S3) were added, with irrigation every 5 days for a total of three applications. For each salt concentration, a control (NaCl) group was set up by applying salt alone without adding silicon, with the NaCl control group being CK. Each treatment included three biological replicates; each replicate contained 9 individual potted plants. Plant growth-related traits and antioxidant indices were all carried out using materials obtained from this pot experiment. Samples for all measurements were harvested approximately 15 days after the initiation of stress treatments, when obvious phenotypic differences among treatment groups could be observed. All potted plants were grown in an artificial climate chamber with consistent environmental conditions: 16 h/8 h light/dark photoperiod, 250 μmol m^−2^ s^−1^ photosynthetic photon flux density, 22 ± 2 °C, and 60% relative humidity. During the experiment, the corresponding treatment solutions were supplied as needed according to substrate moisture, and growth parameters were regularly observed and recorded.

### 2.2. Phenotypic and Root Trait Measurements

Plant height was measured with a ruler, and stem diameter with a vernier caliper. The whole-plant fresh weight was determined using an electronic balance, after which the samples were killed at 105 °C and dried at 75 °C to constant weight to obtain dry weight. The cleaned roots were scanned with an EPSON Perfection V700/V750 scanner (Epson, Suwa, Japan), and root architecture parameters were analyzed using RHIZO2017a software. Meanwhile, plant growth status and morphological changes were regularly observed and recorded.

### 2.3. Measurement of Physiological-Biochemical Indicators Related to Oxidative-Antioxidant Homeostasis

Phenotypic traits, root morphological parameters, DAB and NBT histochemical staining, SOD, POD, CAT activities, chlorophyll content, MDA, proline, and leaf relative water content were determined using in vitro tissue-cultured potato plantlets harvested at 28 days post-inoculation. For potted potato seedlings sampled at 15 days after salt and Si treatment, we measured phenotypic traits, root length, fresh and dry biomass, SOD, POD, CAT activities, MDA, proline, leaf relative water content, and SPAD value.

To evaluate salt-triggered oxidative stress and the antioxidant-regulating effects of exogenous silicon, we determined reactive oxygen species (ROS) accumulation, antioxidant-enzyme activities, membrane lipid peroxidation, osmotic-adjustment substances, leaf relative water content, and photosynthetic pigment parameters. In situ histochemical staining was performed using 3,3′-diaminobenzidine (DAB) and nitroblue tetrazolium (NBT) to visualize in situ hydrogen peroxide (H_2_O_2_) and superoxide anion (O_2_^−^) accumulation in potato leaves, respectively [37]. The activities of three core antioxidant enzymes, superoxide dismutase (SOD), peroxidase (POD), and catalase (CAT), as well as malondialdehyde (MDA) content, were assayed via standard spectrophotometric protocols (nitroblue tetrazolium, guaiacol, and ultraviolet absorption methods for SOD, POD, and CAT, respectively; thiobarbituric acid reaction for MDA) [38,39]. Free proline content was determined using the acidic ninhydrin color-reaction method [40]. Relative electrical conductivity (REC), representing cell-membrane injury, was determined by comparing the electrical conductivity of leaf leachate before and after boiling-water bath treatment [41]. Crude enzyme extracts for all antioxidant-related assays were freshly prepared, and all biochemical measurements were conducted with three independent biological replicates.

Subsequently, photosynthetic pigments were extracted using 80% acetone and quantified by spectrophotometry. Relative water content (RWC) was measured on the third-to-fourth fully expanded healthy leaves at 15 days post-treatment, and calculated using the formula: [(fresh weight − dry weight)/(turgid weight − dry weight)] × 100%. Cell-membrane integrity was assessed by measuring relative electrical conductivity (REC), by comparing the electrical conductivity of leaf leachate before and after boiling treatment [42].

### 2.4. Transcriptome Sequencing

All transcriptome analyses described in Section 2.4, Section 2.5, Section 2.6, Section 2.7 and Section 2.8 were performed using in vitro-grown tissue-cultured plantlets of potato cultivar ‘Atlantic’. Tissue-cultured plantlets of the cultivar ‘Atlantic’ were inoculated onto culture medium and cultured in closed vessels in a tissue culture room under the following conditions: a photoperiod of 16 h light/8 h dark, a photosynthetic photon flux density of 250 μmol m^−2^ s^−1^, a temperature of 22 ± 2 °C, and a relative humidity of 60%. At 28 days post-treatment, three experimental groups, including control (CK), 0.3% NaCl, and 0.3% NaCl supplemented with 1 mM Si, were harvested. Shoots and roots were separately collected from each treatment, immediately frozen in liquid nitrogen, and stored at −80 °C until RNA extraction. Transcriptome sequencing was subsequently performed by OE Biotech Co., Ltd. (Shanghai, China) using the Illumina NovaSeq platform (Illumina Inc., San Diego, CA, USA), with three biological replicates per treatment.

### 2.5. Total RNA Extraction, cDNA Library Construction, and Transcriptome Sequencing

Total RNA was extracted from samples using RNeasy Plant Mini Kit (QIAGEN, Hilden, Germany), and its purity and integrity were assessed by agarose gel electrophoresis and NanoDrop 2000 micro-spectrophotometry (Thermo Fisher Scientific, Wilmington, DE, USA). Qualified RNA was used to construct a cDNA library, which was subsequently subjected to paired-end transcriptome sequencing on the Illumina NovaSeq platform (Illumina Inc., San Diego, CA, USA). After removal of adapter sequences and low-quality reads through quality control filtering, the resulting clean reads were aligned to the potato reference genome. Gene expression levels were finally normalized and quantified as FPKM values.

### 2.6. Identification of Differentially Expressed Genes and Functional Enrichment

Differentially expressed genes (DEGs) were identified using the DESeq2 R package with thresholds of |log_2_ fold change| ≥ 1 and false discovery rate-adjusted *p* < 0.05. Venn diagrams were generated to visualize shared and unique DEGs across the comparison groups. Gene Ontology (GO) enrichment and Kyoto Encyclopedia of Genes and Genomes (KEGG) pathway analyses were subsequently performed on all identified DEGs. Significantly enriched biological processes and metabolic pathways were visualized as bubble charts, with an FDR < 0.05 as the threshold for significance.

### 2.7. Weighted Gene Co-Expression Network Analysis (WGCNA)

Weighted gene co-expression network analysis (WGCNA) was constructed using the WGCNA R package based on the normalized expression matrix with an optimal soft-thresholding power selected to satisfy scale-free topology. An unsigned network was built with a minimum module size of 50 genes. Module eigengenes were correlated with phenotypic traits, and genes with module membership > 0.8 and gene significance > 0.7 were considered candidate hub genes.

### 2.8. Quantitative Real-Time PCR Analysis

The qPCR reaction was performed in a total volume of 20 μL, consisting of 10 μL of 2× SYBR Green II Master Mix (Takara-Bio, Kusatsu, Shiga, Japan), 1 μL of cDNA, 2.5 μL each of gene-specific primers (10 μmol/L), and 6.5 μL of ddH_2_O. The thermal cycling conditions were as follows: initial denaturation at 95 °C for 30 s, followed by 39 cycles of 95 °C for 5 s and 60 °C for 30 s. Primers were designed using the Primer 3 online tool (https://primer3.ut.ee), and their sequences are listed in Table S1. *StActin* was used as the internal reference gene in potato. Each sample was analyzed with three biological replicates and three technical replicates, and relative gene expression levels were calculated using the 2^−ΔΔCt^ method.

### 2.9. Statistical Analysis

Data obtained from three independent biological replicates were presented as means ± standard deviation (SD). Statistical analyses were performed using SigmaPlot 10.1 software. Two-tailed Student’s *t*-test was used for comparisons between two groups, while one-way analysis of variance (ANOVA) followed by Tukey’s post hoc test was applied for multiple-group comparisons. Differences were considered statistically significant at *p* < 0.05 and highly significant at *p* < 0.01.

## 3. Result

### 3.1. Screening Silicon Concentrations for Phenotypic Performance In Vitro

**Figure 1:**
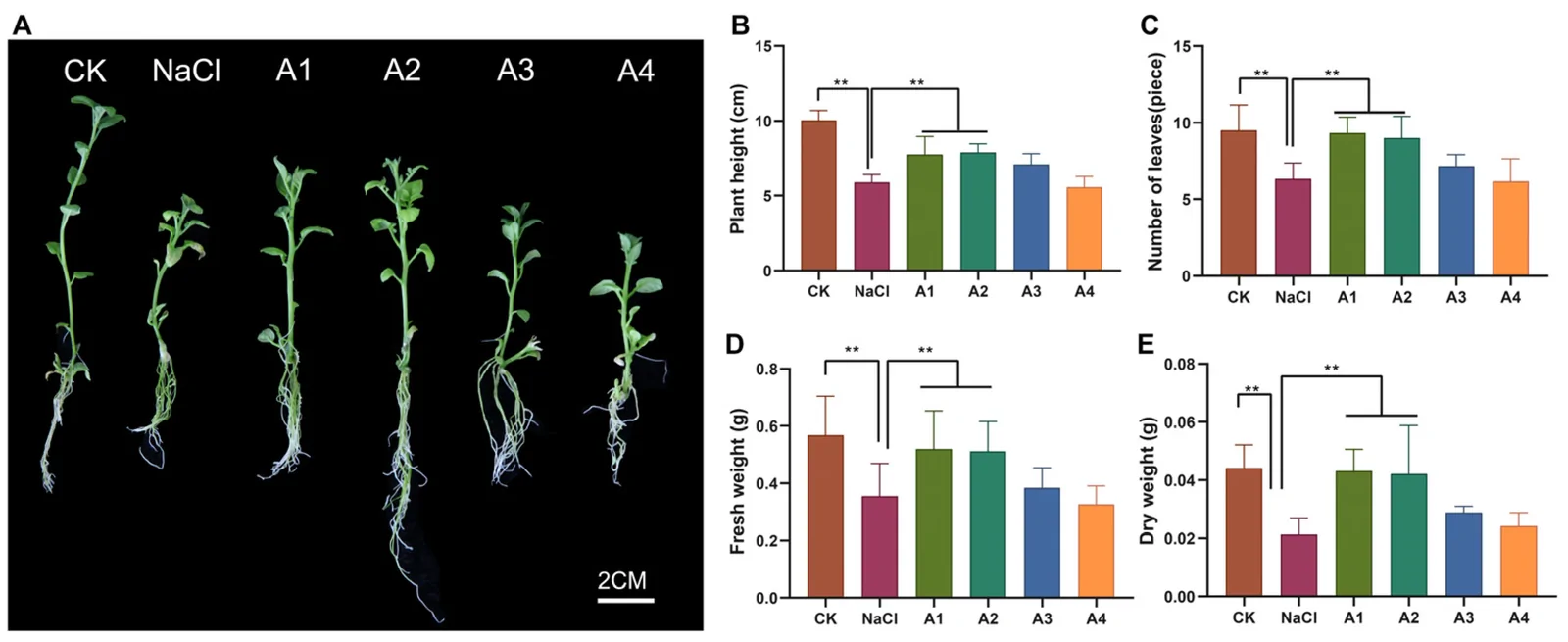
Effects of exogenous silicon at different concentrations on the growth performance of potato tissue-cultured plantlets under salt stress. (**A**) Representative growth phenotypes of plantlets. Scale bar = 2 cm. (**B**) Plant height. (**C**) Leaf number. (**D**) Fresh weight. (**E**) Dry weight. CK: control; NaCl: salt-stress treatment; A1, A2, A3, and A4 represent salt-stressed plantlets (0.3% NaCl) supplied with 0.5, 1.0, 2.0, and 3.0 mM silicon, respectively. Data are presented as mean ± standard deviation (SD). ** indicates statistically significant difference at *p* < 0.01.

Growth parameters (plant height, number of fresh leaves, and dry weight) directly reflect the extent of salt-induced damage and the alleviative effects of silicon. As shown in Figure 1, NaCl significantly inhibited the growth of tissue-cultured plantlets, with all indicators being markedly lower than those of the CK. Following silicon application, all parameters first increased and then decreased with rising Si concentration. Compared with the single NaCl treatment, both A1 and A2 Si concentrations significantly improved plant growth. Notably, A2 exhibited the optimal alleviative effect, restoring the fresh and dry weights to levels close to those of the CK group. Under A4, the increases in all parameters declined, even falling slightly below those of A2, suggesting that excessive silicon might exert a slight inhibitory effect.

### 3.2. Root Traits of Tissue-Cultured Plantlets Under Different Silicon Regimes

**Figure 2:**
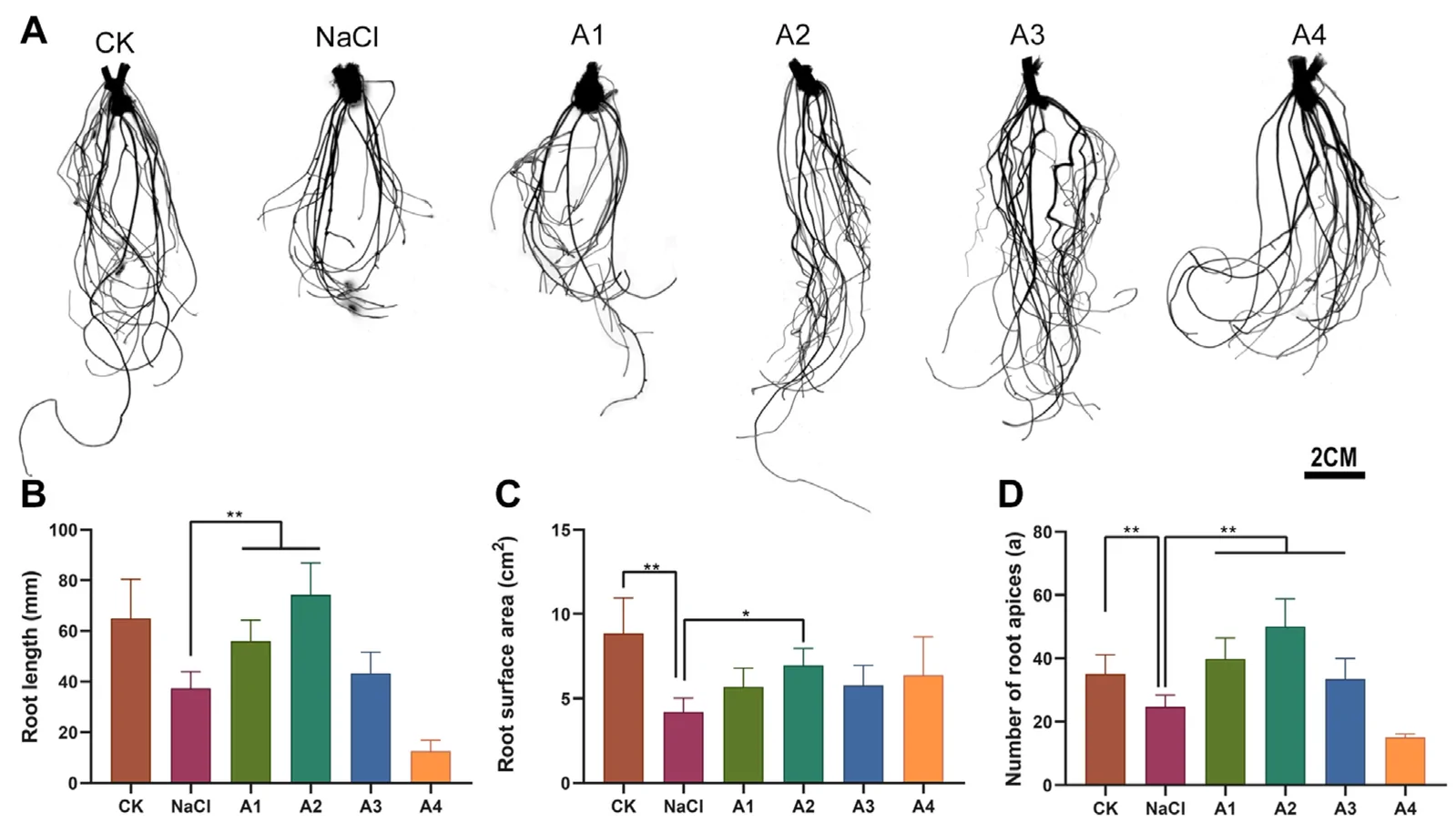
Effects of different exogenous silicon concentrations on root morphology of potato tissue-cultured plantlets under salt stress. (**A**) Representative root system phenotypes. Scale bar = 2 cm. (**B**) Root length. (**C**) Root surface area. (**D**) Number of root apices. CK: control; NaCl: salt-stress treatment; A1, A2, A3, and A4 represent salt-stressed plantlets (0.3% NaCl) supplied with 0.5, 1.0, 2.0, and 3.0 mM silicon, respectively. Data are presented as mean ± standard deviation (SD). * and ** denote significant differences at *p* < 0.05 and *p* < 0.01, respectively.

Root morphological traits including root length, root surface area, and root-tip number reliably reflect salt-stress-induced root growth inhibition as well as the mitigating effect of exogenous silicon. As illustrated in Figure 2, NaCl stress markedly suppressed root development, with all root-related indices significantly reduced relative to the CK group. Upon silicon supplementation, these root parameters exhibited a concentration-dependent trend of rising first and then declining. The A2 treatment yielded the highest values for root length, root surface area, and root-tip number; these indices were significantly greater than those of the NaCl group, and root length and root surface area recovered to levels close to the CK. Moderate improvements were also observed under A1 and A3, though their promotive effects were weaker than A2. By contrast, A4 caused a drop in all root traits, even somewhat below the performance of A2, implying that excess silicon may impose mild growth inhibition. Collectively, A2 represented the optimal concentration for alleviating salt-mediated repression of potato root growth.

### 3.3. Silicon-Mediated Modulation of Oxidative Stress and Antioxidant Defense Under Salt Stress

**Figure 3:**
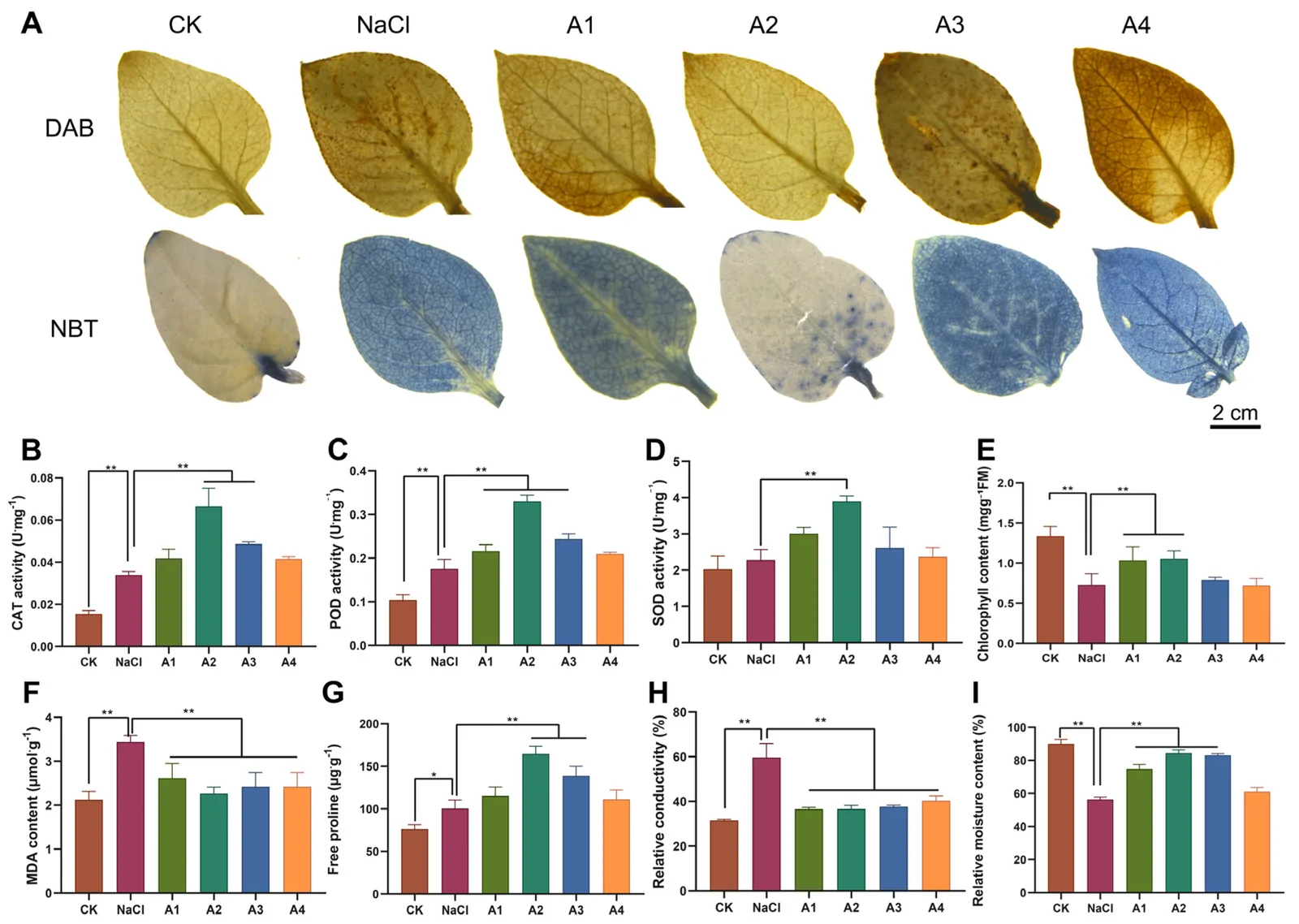
Effects of exogenous silicon on reactive oxygen species accumulation, antioxidant enzyme activities, and physiological traits of potato leaves under salt stress. (**A**) In situ DAB and NBT histochemical staining for H_2_O_2_ and O_2_^−^; scale bar = 2 cm. (**B**–**I**) Quantitative determination of CAT activity, POD activity, SOD activity, chlorophyll content, MDA content, free proline content, relative electrical conductivity, and relative moisture content. CK: control; NaCl: salt-stress treatment; A1, A2, A3, and A4 represent salt-stressed plantlets (0.3% NaCl) supplied with 0.5, 1.0, 2.0, and 3.0 mM silicon, respectively. Data are presented as mean ± SD. * and ** indicate significant differences at *p* < 0.05 and *p* < 0.01, respectively.

Histochemical DAB and NBT staining revealed severe reactive oxygen species (ROS) burst in salt-stressed potato leaves. Compared with the CK, NaCl treatment dramatically elevated the in situ accumulation of H_2_O_2_ and O_2_^−^, evidenced by intense brown and blue staining, respectively (Figure 3A). Exogenous silicon reduced ROS staining intensity in a concentration-dependent way. The 1.0 mM Si (A2) group displayed the faintest staining signal; its CAT, POD, and SOD activities increased by 1.94-fold, 1.71-fold, and 1.68-fold relative to the NaCl-only group, representing the strongest induction of enzymatic antioxidant capacity among all Si treatments (Figure 3B–D). Lower (A1) and higher (A3) silicon concentrations also significantly up-regulated antioxidant enzyme activities, yet their effects were weaker than those of A2. At the highest Si concentration (A4), antioxidant enzyme activities declined, and leaf ROS staining rebounded, demonstrating that excessive silicon failed to sustain the enhanced antioxidant response and could not further relieve oxidative damage.

Beyond oxidative-stress alleviation, Si rescued salt-triggered physiological impairments (Figure 3E–I). Salt stress decreased chlorophyll content by ~45% relative to CK, while A2 recovered pigment levels to ~78% of CK values. Salt-induced membrane damage was evidenced by 2.8-fold higher MDA and 2.2-fold higher REC; A2 reduced MDA by ~55% and REC by ~40% compared with NaCl-only plants. Salt stress increased proline ~3.5-fold, and Si further elevated proline in a concentration-dependent pattern, peaking at 1.4-fold over NaCl in A2, which strengthened osmotic adjustment. In summary, 1.0 mM Si (A2) was the optimal dose to protect membranes, maintain photosynthesis, and improve osmotic homeostasis for salt-stressed potato plantlets.

### 3.4. Silicon-Regulated Growth, Oxidative-Antioxidant Responses Under Low-Salt Stress

**Figure 4:**
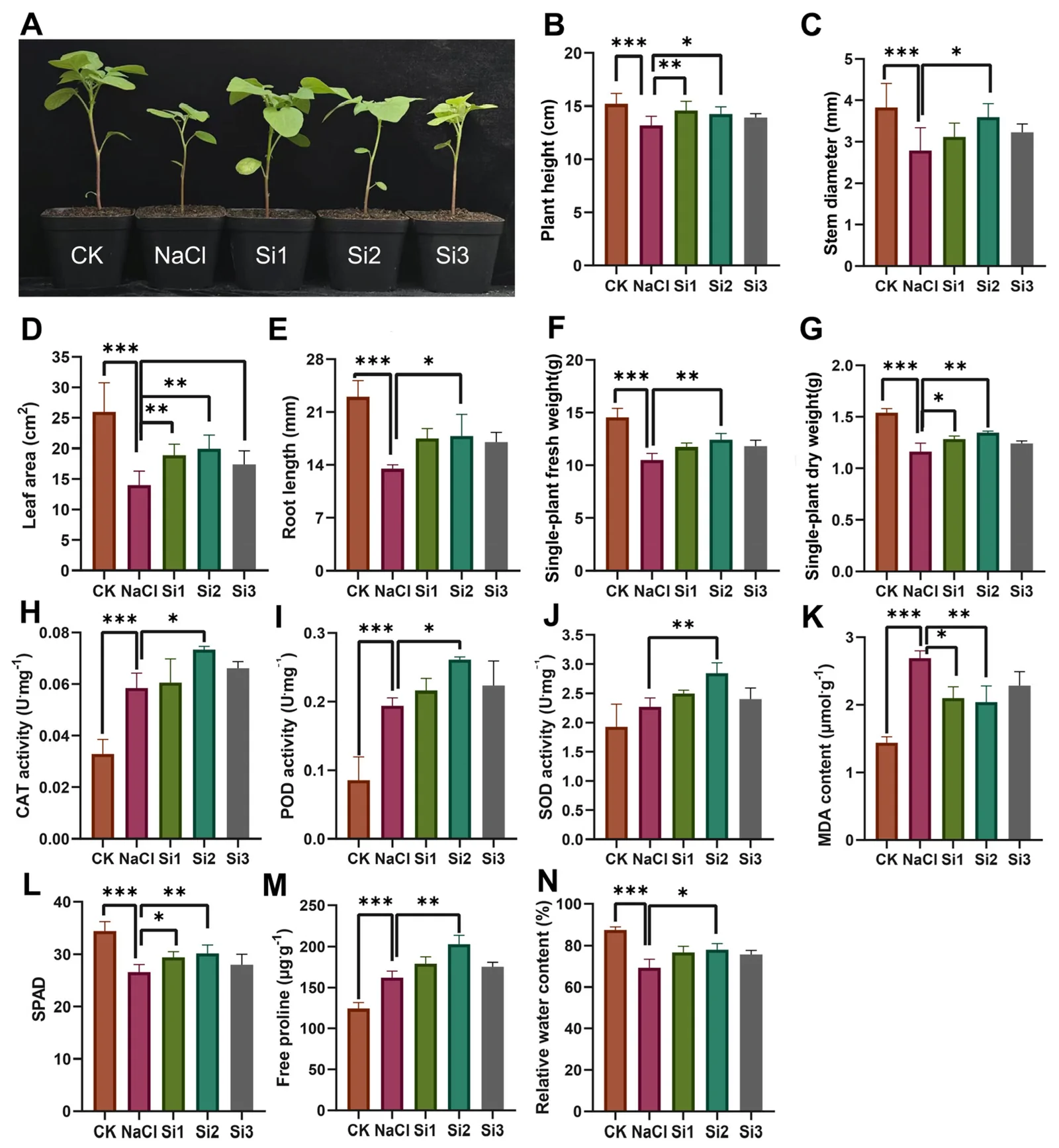
Growth and physiological responses of potted potato seedlings to gradient exogenous silicon under low-salinity stress (100 mM). (**A**) Representative plant phenotypes. (**B**–**G**) Quantification of plant height, stem diameter, leaf area, root length, single-plant fresh weight, and single-plant dry weight. (**H**–**N**) CAT activity, POD activity, SOD activity, MDA content, SPAD value, free proline content, and relative water content. CK: non-stressed control; NaCl: low-salinity stress alone; Si1, Si2, and Si3: low salinity-stressed seedlings (100 mM NaCl) supplied with 1, 2, and 3 mM silicon, respectively. Data are presented as mean ± standard deviation (SD). *, **, and *** denote significant differences at *p* < 0.05, *p* < 0.01, and *p* < 0.001, respectively.

To further verify the mitigating effect of silicon observed in MS-cultured plantlets, subsequent experiments were performed using potted seedlings under low-salinity stress. Low-salinity stress suppressed multiple growth traits including plant height, stem diameter, leaf area, root length, and single-plant fresh and dry weight (Figure 4A–G). Upon silicon supplementation, these growth indices exhibited a concentration-dependent trend of rising first and then declining. The Si2 treatment yielded the highest values for all measured parameters; these were significantly greater than those of the NaCl group, and most traits recovered to levels close to the CK. Si1 also mitigated salt-induced growth inhibition, though its promotive effect was weaker than Si2. In contrast, Si3 led to reduced performance across all growth indices, with several parameters even lower than the NaCl group, implying growth repression triggered by excessive silicon. Biomass accumulation followed the same tendency as shoot-related traits, with Si2 showing the best performance, which identified Si2 as the optimal dosage for alleviating low-salinity stress.

Low-salt stress triggered obvious oxidative damage and activated the antioxidant defense system in potato leaves. Exogenous silicon differentially modulated the activities of antioxidant enzymes. CAT activity was significantly increased by Si1 and Si2, reaching its maximum under Si2 (1.95-fold higher than the NaCl group), before declining at Si3. POD activity showed limited variation across all treatments, while SOD activity gradually decreased with rising silicon concentration, falling to the lowest level in Si2. Consistent with enhanced antioxidant protection, MDA content, a marker of membrane lipid peroxidation and oxidative injury, was significantly reduced by Si2 compared with the NaCl-stressed control (Figure 4H–K).

Other stress-related physiological parameters also responded to silicon addition (Figure 4L–N). Free proline was further accumulated in Si1 and Si3 groups, whereas proline level under Si2 was similar to that of NaCl-only plants. All silicon-treated seedlings maintained significantly higher leaf relative water content, which helped sustain cellular water homeostasis under salt challenge. Taken together, these results demonstrated that optimal silicon concentration (Si2) improved salt tolerance mainly by strengthening antioxidant enzyme-mediated ROS-scavenging capacity, thereby reducing oxidative-triggered membrane damage.

### 3.5. Silicon-Mediated Growth Performance and Oxidative-Antioxidant Responses Under High-Salt Stress

To further explore whether the silicon-induced salt-tolerance effect observed under low-salinity conditions persists at more severe salt levels, potted potato seedlings were exposed to 200 mM NaCl for high-salinity validation experiments.

**Figure 5:**
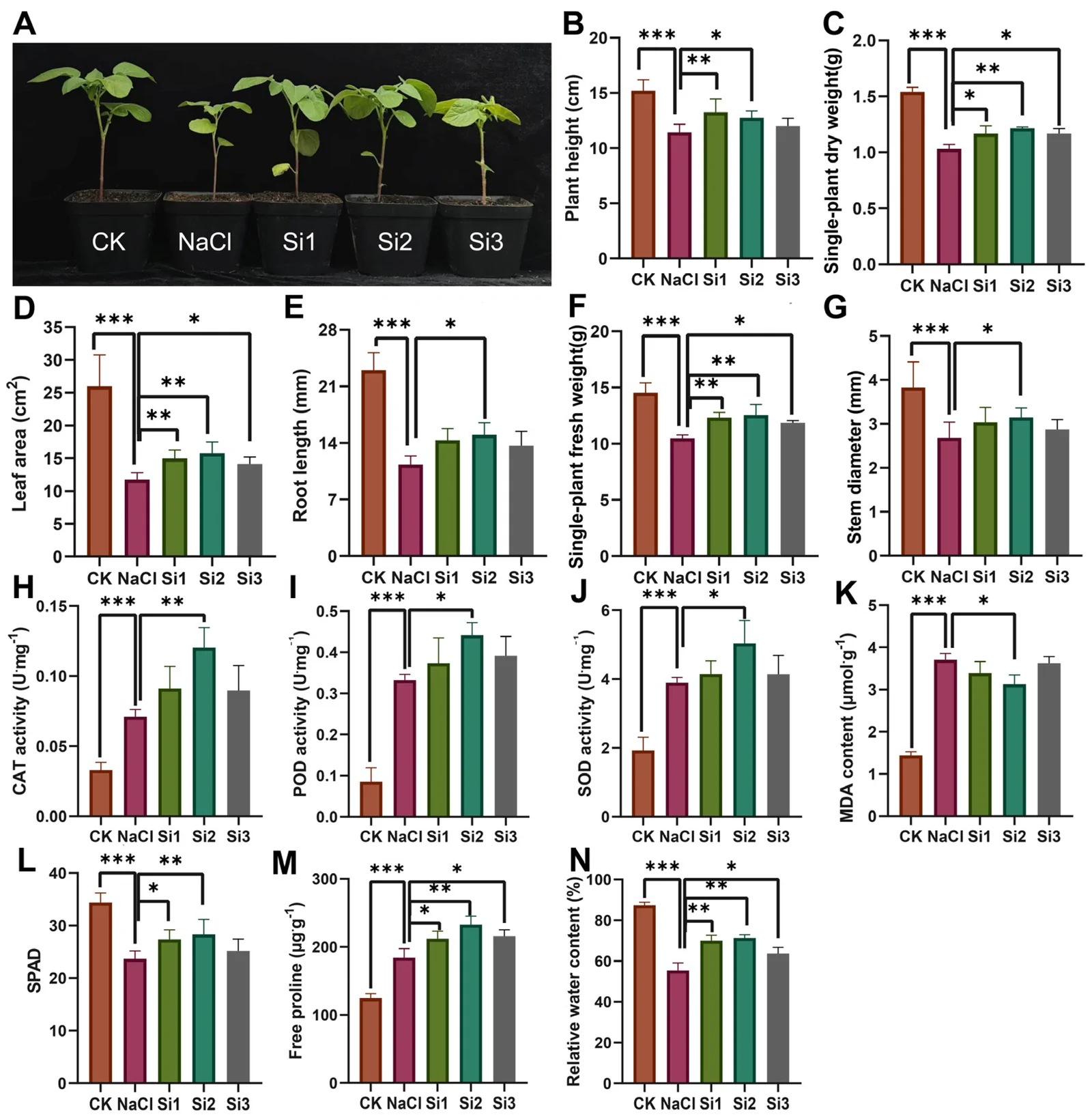
Growth and physiological responses of potted potato seedlings to gradient exogenous silicon under high-salinity stress (200 mM). (**A**) Representative plant phenotypes. (**B**–**G**) Plant height, single-plant dry weight, leaf area, root length, single-plant fresh weight, and stem diameter. (**H**–**N**) CAT activity, POD activity, SOD activity, MDA content, SPAD value, free proline content, and relative water content. CK: non-stressed control; NaCl: high-salinity stress alone; Si1, Si2, and Si3: low-salinity-stressed seedlings (200 mM NaCl) supplied with 1, 2, and 3 mM silicon, respectively. Data are presented as mean ± SD. *, **, and *** denote significant differences at *p* < 0.05, *p* < 0.01, and *p* < 0.001, respectively.

High-salinity stress caused severe growth inhibition across all above-ground and root-related parameters. Compared with CK, NaCl alone reduced plant height by 24.7%, leaf area by 54.2%, and single-plant dry weight by 31.1% (Figure 5A–G). After exogenous silicon application, all growth-related indicators exhibited a typical concentration-dependent trend, first increasing and then declining. The Si2 treatment achieved the maximum values for every growth trait; its plant height and dry weight were increased by 18.3% and 14.6%, respectively, relative to the NaCl-only group, yet most growth parameters were only partially restored and failed to recover to control levels. Si1 produced a weaker protective effect compared with Si2. By contrast, Si3 triggered a reduction in all growth indices, confirming that excessive silicon exerted mild inhibitory effects even under severe salt-stress conditions. Consistent with shoot traits, whole-plant fresh- and dry-weight biomass peaked in the Si2 group, identifying Si2 as the optimal silicon dosage for high-salt mitigation.

High-salinity stress activated the plant antioxidant defense system, and exogenous silicon further modulated ROS-scavenging enzyme activities in a concentration-dependent manner. The activities of three core antioxidant enzymes (CAT, POD, and SOD) gradually rose with increasing silicon supply and reached their highest levels in the Si2 treatment. Relative to the NaCl group, CAT activity in Si2 was elevated 2.1-fold, POD activity increased by 34.4%, and SOD activity rose by 28.9%, demonstrating that suitable silicon supplementation could strengthen enzymatic antioxidant capacity to counteract salt-provoked oxidative damage. MDA content, an indicator of membrane lipid peroxidation, did not show obvious differences between NaCl-stressed and silicon-treated seedlings (Figure 5H–K). Additionally, SPAD-based chlorophyll readings and free proline concentrations exhibited limited variation across treatments (Figure 5L–N). These findings implied that, under severe high-salinity stress, silicon-conferred salt tolerance was primarily achieved via enhancement of antioxidant enzyme-driven ROS scavenging, rather than adjustments to membrane lipid peroxidation, photosynthetic pigment stability, or proline-related osmotic regulation.

### 3.6. Transcriptome Profiling of Silicon-Modulated Salt-Tolerance and Antioxidant-Related Responses

**Figure 6:**
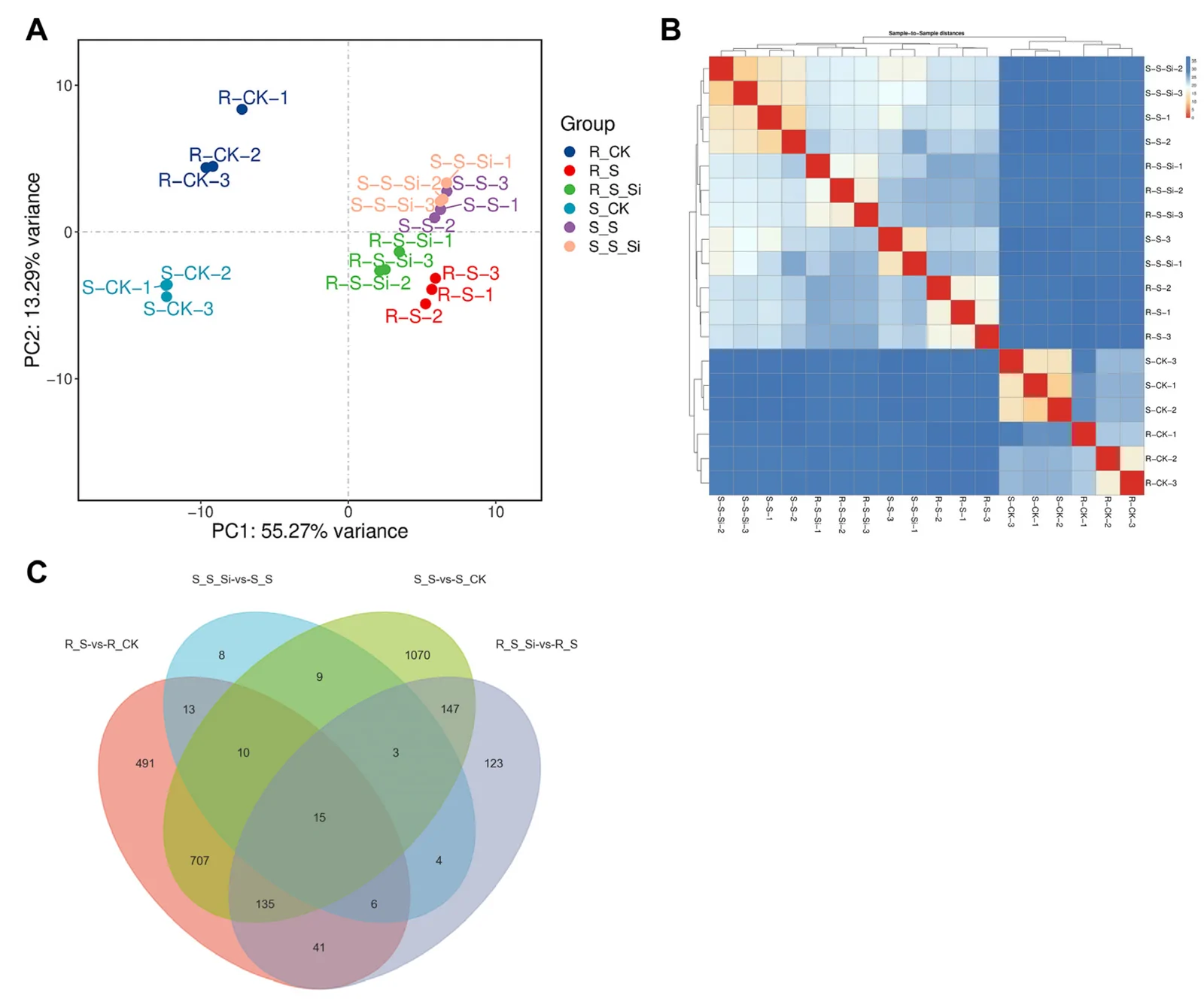
Global transcriptome profiling of potato root and stem tissues under salt stress with silicon application. (**A**) Principal component analysis (PCA) of all transcriptome samples. (**B**) Sample-to-sample distance heatmap based on Euclidean distance. (**C**) Venn diagram illustrating the overlap of differentially expressed genes from four pairwise comparisons. R-CK/R-S/R-S-Si: root samples under control, salt stress, salt plus silicon; S-CK/S-S/S-S-Si: stem samples under corresponding treatments.

The phenotypic and physiological data obtained from both in vitro and pot experiments consistently demonstrated that exogenous silicon effectively alleviates salt-induced growth inhibition and oxidative damage in potato, primarily through enhancing antioxidant enzyme activities (SOD, POD, CAT), reducing ROS accumulation and membrane lipid peroxidation, and maintaining osmotic balance. These findings strongly suggest that Si orchestrates a multifaceted protective response at the physiological level. However, the underlying molecular regulatory network, particularly the key genes and signaling pathways through which Si coordinates antioxidant defense, osmotic adjustment, and other stress-responsive processes, remains largely unclear. To address this gap, we performed comparative transcriptome sequencing on roots and shoots from three representative treatments: control (CK), salt stress alone (NaCl), and salt stress combined with 1.0 mM Si (NaCl + Si), using plant materials derived from in vitro tissue-culture experiments, with three biological replicates per treatment. Principal component analysis (PCA) showed that PC1 and PC2 explained 55.27% and 13.29% of total variance, with a cumulative contribution of 68.56%, capturing major transcriptomic variation across samples (Figure 6A). Tight clustering of biological replicates reflected good experimental reproducibility. Euclidean-distance-based sample-to-sample distance heatmap further supported this observation, with low within-group distances across replicates (Figure 6B). Consistent with PCA, both salt stress and silicon supplementation substantially reshaped transcript profiles in roots and stems, validating the reliability of RNA-seq data for downstream differential-gene and enrichment analyses.

Venn diagram analysis revealed prominent tissue-specific transcriptional responses under salt stress: 1070 DEGs were stem-specific, 491 DEGs were root-specific, and 707 DEGs were shared between tissues (Figure 6C). Upon silicon treatment, only 8 unique DEGs were identified in stems, whereas 123 unique DEGs were detected in roots. Only 15 genes overlapped across all four comparisons, indicating that salt- and silicon-triggered transcriptomic reprogramming in stems and roots is largely governed by tissue-specific regulatory pathways rather than universal core genes.

### 3.7. Volcano Plot Analysis of Differentially Expressed Genes

Volcano plot analysis illustrated distinct salt-triggered transcriptomic alterations in potato roots and stems, which were substantially reprogrammed upon exogenous silicon supply (Figure S1). In stems, salt stress provoked massive transcriptional changes, with 4954 up-regulated (1371 significant) and 4283 down-regulated (725 significant) DEGs. Silicon supplementation sharply diminished the number of salt-responsive DEGs to merely 25 up-regulated and 43 down-regulated genes, indicating silicon mainly dampens salt-triggered transcriptional overactivation instead of initiating new regulatory cascades. Roots exhibited comparable yet more pronounced responses: salt stress induced 1951 up-regulated (961 significant) and 1369 down-regulated (457 significant) DEGs. Silicon reversed this salt-induced transcriptional signature, where down-regulated DEGs (1381, 404 significant) greatly exceeded up-regulated counterparts (636, 70 significant). Taken together, exogenous silicon largely functions to restore salt-disrupted transcript profiles toward homeostatic status.

### 3.8. KEGG Enrichment Analysis of Differentially Expressed Genes

**Figure 7:**
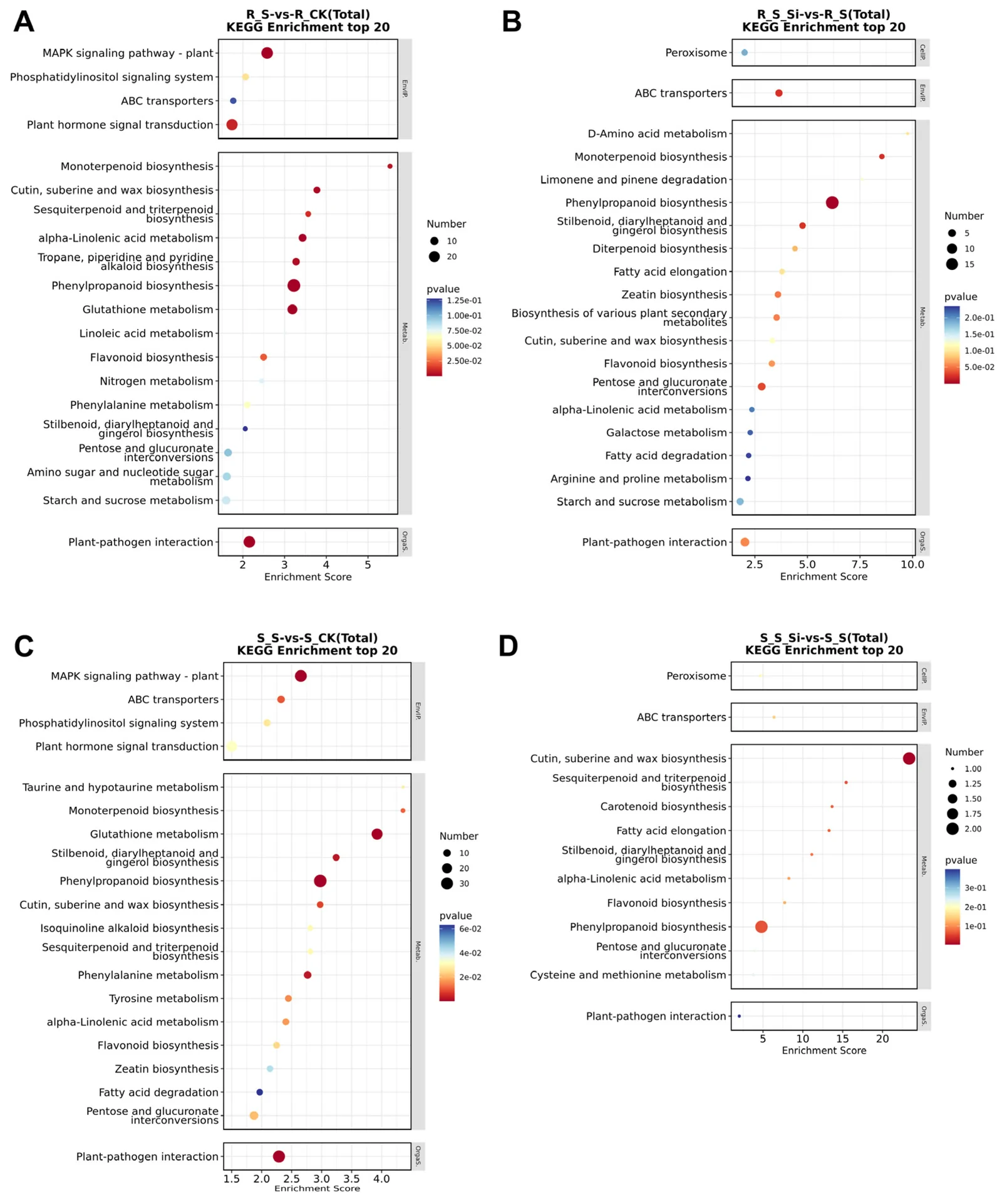
KEGG pathway enrichment scatter plots of differentially expressed genes. (**A**) Root: R_CK vs. R_S; (**B**) root: R_S vs. R_S_Si; (**C**) stem: S_CK vs. S_S; (**D**) stem: S_S vs. S_S_Si. The top 20 enriched KEGG pathways for each comparison are presented. Dot size corresponds to the number of DEGs assigned to each pathway, and color gradient reflects enrichment significance (*p*-value).

KEGG enrichment analysis uncovered distinct oxidative-antioxidant pathway reprogramming in roots and stems under salt stress and silicon treatment (Figure 7). In salt-stressed roots, DEGs were prominently enriched in mitogen-activated protein kinase (MAPK) signaling and plant-hormone signal transduction, alongside pronounced activation of phenylpropanoid, flavonoid, terpenoid, and cutin–suberin biosynthesis pathways closely linked to secondary-metabolite-mediated antioxidant defense, indicative of stress-triggered defensive metabolic remodeling. Upon silicon supplementation, enrichment of these salt-responsive defense pathways was attenuated. Instead, DEGs converged on peroxisome-associated ROS-scavenging pathways, carbohydrate metabolism, fatty-acid β-oxidation, and proline metabolism, which support energy homeostasis, osmotic adjustment, and cellular redox balance. Meanwhile, enhanced enrichment of ABC transporters and D-amino-acid metabolism pointed to improved ion compartmentation and nitrogen turnover that further alleviate salt-provoked oxidative injury.

In stems, salt stress moderately activated MAPK cascades and basal phenylpropanoid pathways. Silicon treatment further boosted enrichment of peroxisome, cutin–suberin, phenylpropanoid, flavonoid, and carotenoid biosynthesis pathways; flavonoids and carotenoids function as critical non-enzymatic antioxidants to scavenge excess ROS, thus strengthening cell-wall barrier capacity and antioxidant protection against oxidative damage. Overall, silicon-conferred salt tolerance displayed evident tissue-specific features: roots mainly maintain metabolic-energy homeostasis and ROS clearance, whereas stems reinforce cell-wall structural protection and non-enzymatic antioxidative defenses, jointly contributing to improved salt adaptability.

### 3.9. Construction of Weighted Gene Co-Expression Network and Identification of Key Modules

Weighted gene co-expression network analysis (WGCNA) was performed to dissect gene modules linked to salt-stress physiological traits. A suitable soft-thresholding power was chosen to satisfy scale-free topology (scale-free fit index R^2^ > 0.8) while maintaining reasonable mean connectivity, supporting reliable module detection. Hierarchical clustering combined with the DynamicTreeCut algorithm identified 16 distinct co-expression modules from transcriptome profiles (Figure S2A).

Module-trait relationships were evaluated by Pearson correlation between module eigengenes and seven physiological indices (CAT, POD, SOD, MDA, free proline, relative water content, relative electrical conductivity). Multiple modules displayed strong trait associations (Figure S2B,C). The MEblue module had prominent negative correlations with CAT, POD, SOD, and relative conductivity (*r* = −0.71, −0.65, −0.60, −0.75; *p* < 0.01). MElightgreen showed positive correlations with CAT, POD, SOD, and free proline (*r* = 0.74, 0.75, 0.66, 0.57; *p* < 0.01). MEturquoise was positively correlated with CAT, POD, and relative conductivity (*r* = 0.67, 0.63, 0.73; *p* < 0.01). MEroyalblue correlated positively with MDA and relative conductivity (*r* = 0.60, 0.72; *p* < 0.01). These four modules were retained as key candidate modules for subsequent analysis.

### 3.10. WGCNA Hub Gene Screening, qRT-PCR Validation, and Regulatory Model Construction

**Figure 8:**
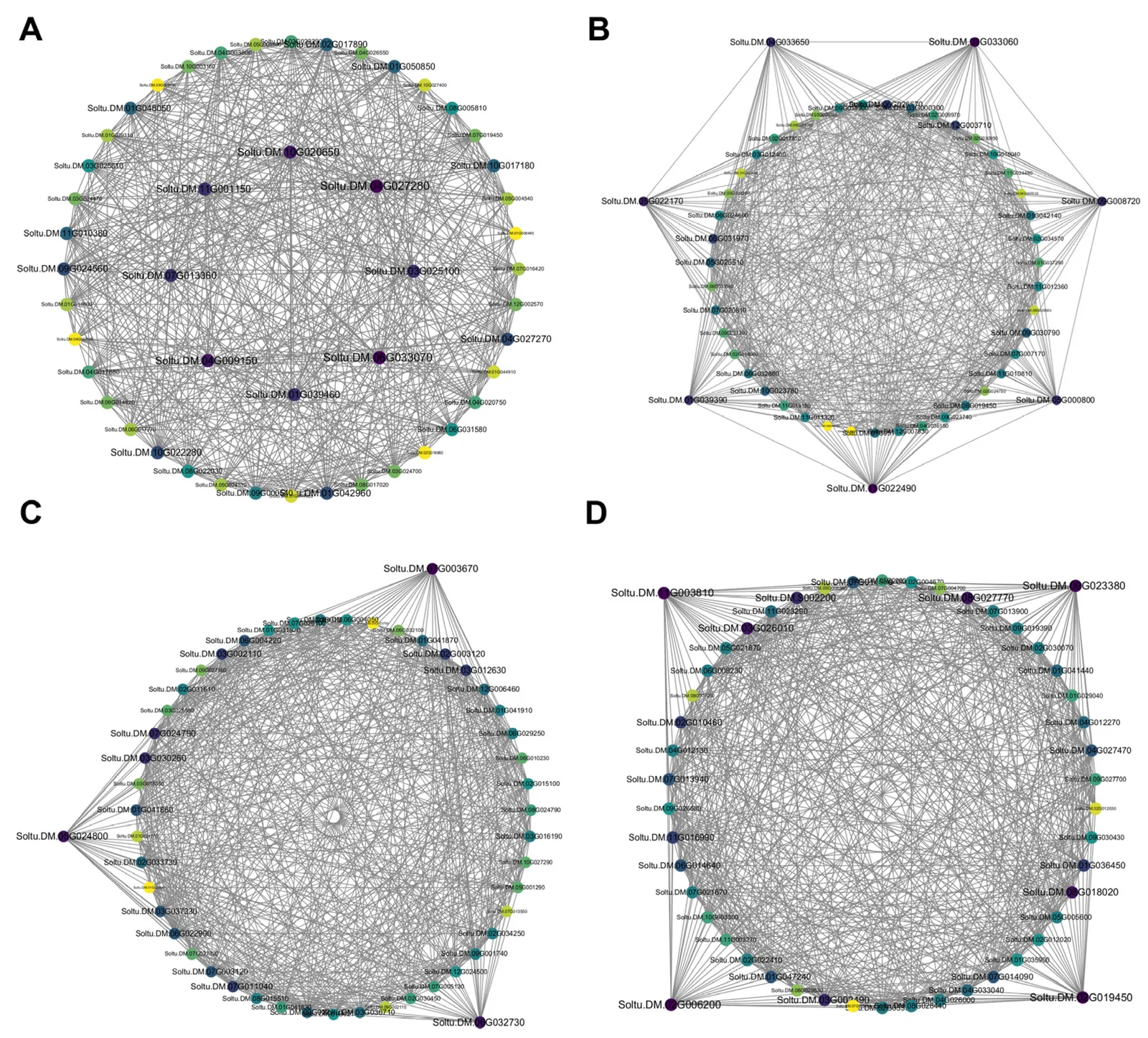
Co-expression network visualization and hub-gene identification for four candidate WGCNA modules. (**A**) MEblue module; (**B**) MElightgreen module; (**C**) MEroyalblue module; (**D**) MEturquoise module.

To explore the inner-module architecture of the four candidate modules, their co−expression networks were visualized via Cytoscape v3.9.1 (Cytoscape Consortium, San Diego, CA, USA), and hub genes were identified according to gene connectivity degree (Figure 8A–D, Table S1). In total, 11, 7, 5, and 7 hub genes were obtained from the MEblue, MElightgreen, MEroyalblue, and MEturquoise modules, respectively. Gene functional annotations were acquired from Spud DB and NCBI, with homologous functional references derived from *Arabidopsis thaliana* via the TAIR database (Tables S2–S5).

Based on co-expression network analysis, hub genes were identified from four pivotal WGCNA modules (MElightgreen, MEblue, MEroyalblue, and MEturquoise), and their biological functions were annotated via homologous sequence alignment with *Arabidopsis thaliana* (Tables S1–S4). These module-specific hub genes exhibited clearly differentiated functional characteristics, forming distinct regulatory modules for silicon-mediated salt tolerance in potato. The MElightgreen module hub genes (e.g., EF1A, ATP81, OR27, ASS1) primarily govern basal metabolism, translational regulation, and photomorphogenesis, which maintain fundamental cellular metabolic homeostasis and provide the energy basis for stress adaptation. In contrast, the MEblue module enriches core genes associated with RNA post-transcriptional processing, sugar metabolism, and cell-wall stress perception (e.g., ALBA1, PNT1, CORK1, SUS1), functioning in stress signal sensing, post-transcriptional fine-tuning, and carbon resource redistribution under salt stress. The MEroyalblue module is dominated by genes related to hypoxia response, lignin biosynthesis, and abiotic stress defense, including Hb1, PRX52, LEA, and EH1, which facilitate cell-wall reinforcement and alleviate salt-induced cellular damage. Meanwhile, the MEturquoise module contains key hub genes involved in secondary metabolite synthesis, glutathione antioxidant system (GSH1), membrane transport (MRP4), and dehydration protection (DHN), directly empowering ROS scavenging, ion transport, and osmotic adjustment capacity of salt-stressed plantlets.

Collectively, the four candidate modules cover multi-dimensional regulatory pathways, ranging from basic energy metabolism and translational regulation to cell-wall remodeling, ROS-scavenging and antioxidant defense, membrane transport, and osmotic protection. These functionally differentiated hub-gene modules do not work independently, but synergistically construct a systematic regulatory network, in which antioxidant-related pathways occupy a central position for silicon-mediated salt adaptation. It is speculated that these core hub genes serve as critical molecular nodes, integrating multiple physiological and molecular processes including enzymatic and non-enzymatic ROS homeostasis regulation, to drive global transcriptional and physiological reprogramming. This layered and collaborative regulatory mechanism, with coordinated antioxidant responses as a key functional module, ultimately improves the comprehensive salt tolerance of potato seedlings and reveals the molecular basis of exogenous silicon in mitigating salt-induced oxidative damage.

**Figure 9:**
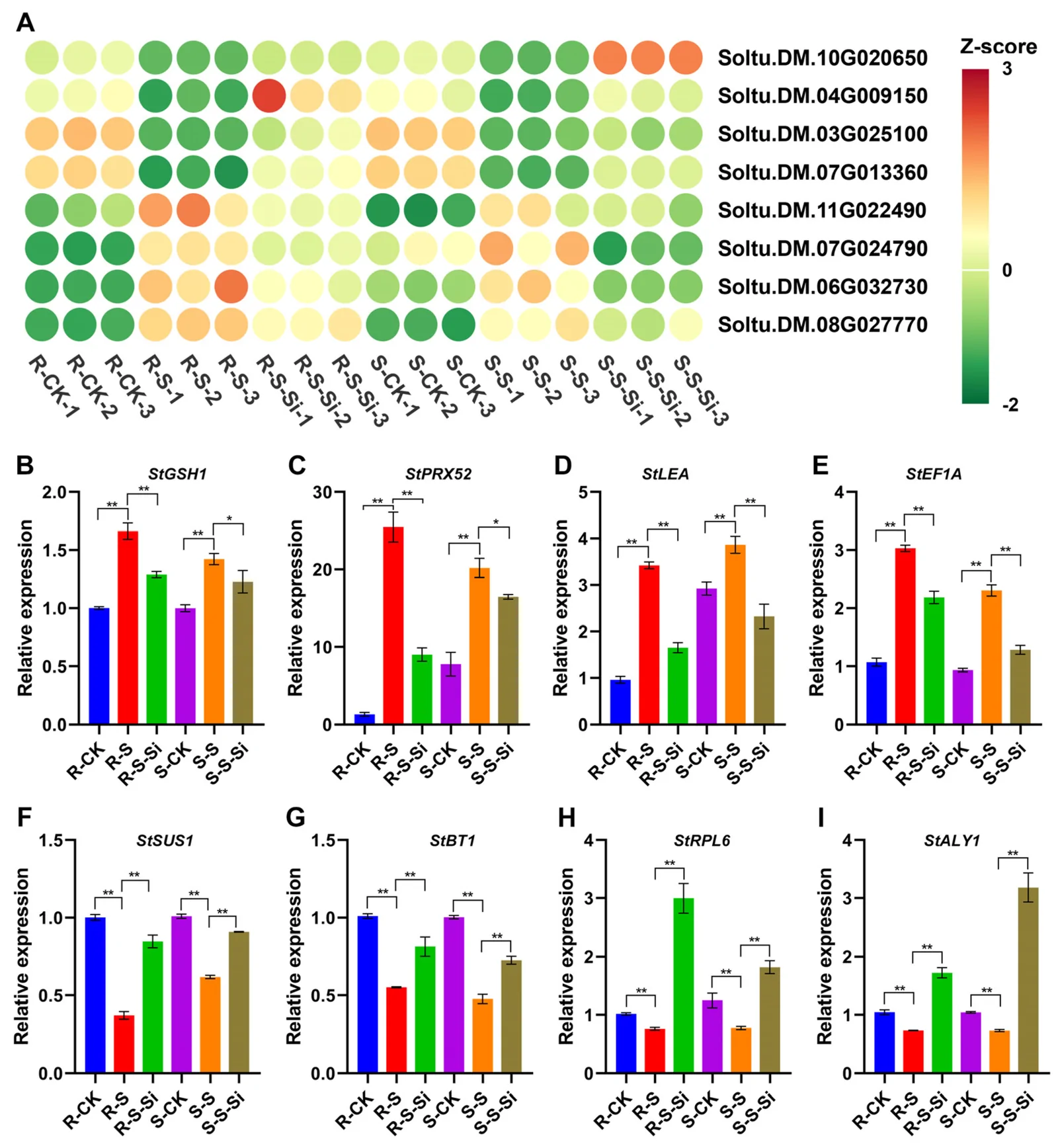
Expression profiles of eight hub-genes under different treatments. (**A**) Heat-map showing transcriptomic FPKM values of the eight hub-genes based on Z-score normalization. (**B**–**I**) RT-qPCR validation of the relative expression levels of *StGSH1*, *StPRX52*, *StLEA*, *StEF1A*, *StSUS1*, *StBT1*, *StRPL6*, and *StALY1*. R, root; S, shoot; CK, control; S, NaCl-only salt-stress treatment; S-Si, salt stress plus exogenous silicon. Data are presented as mean ± standard error (*n* = 3). Significant differences are indicated by asterisks (* *p* < 0.05; ** *p* < 0.01).

To validate the reliability of transcriptomic profiles and WGCNA screening results, eight core hub genes, including *StALY1* (Soltu.DM.10G020650), *StRPL6* (Soltu.DM.04G009150), *StBT1* (Soltu.DM.03G025100), *StSUS1* (Soltu.DM.07G013360), *StEF1A* (Soltu.DM.11G022490), *StLEA* (Soltu.DM.07G024790), *StPRX52* (Soltu.DM.06G032730), and *StGSH1* (Soltu.DM.08G027770), were selected for qPCR quantification. The expression patterns of these candidate genes were highly consistent between qPCR detection and RNA-seq FPKM data across all treatment groups (Figure 9). These findings strongly verify the reproducibility of our transcriptome dataset and further support the dependability of the identified hub genes and module regulatory networks underlying silicon-mediated salt tolerance in potato.

## 4. Discussion

In the present study, our physiological phenotyping data from both in vitro tissue-culture and pot experiments demonstrated that exogenous silicon confers concentration-dependent salt-stress alleviation in potato. Moderate Si concentrations produced optimal protective effects, whereas higher concentrations failed to generate further benefits and even exerted mild growth inhibition. This observed dose–response pattern in potato is consistent with previous reports in GF677 and GN15 rootstocks and nanosilicon-primed rice seedlings [43,44]. The diminishing returns at supra-optimal Si levels may reflect interference with normal cellular metabolism or competition with other nutrients, underscoring the practical need for precision Si fertilization in potato cultivation [45]. This concentration-dependent efficacy also carries important agronomic implications: it suggests that optimal application rates must be empirically determined for specific cultivars and growth systems. It is worth noting that silicon is regarded as a beneficial element for many plant species, rather than an essential mineral nutrient. The fact that our in vitro and pot experiments yielded different optimal concentrations (1.0 mM vs. 2.0 mM) further highlights the influence of growth systems on Si bioavailability. Distinct Si availability exists between solid-soil substrates and liquid-based nutrient solutions or tissue-culture media; multiple environmental factors including pH, ion interactions, substrate adsorption properties, and plant growth status jointly shape Si mobility and the capacity for plant Si uptake. This factor should be carefully considered when extrapolating laboratory findings to field recommendations.

Our physiological measurements in salt-stressed potato tissue-cultured plantlets revealed that Si substantially mitigated salt-provoked oxidative injury by boosting the activities of SOD, POD, and CAT antioxidant enzymes and lowering ROS accumulation and membrane lipid peroxidation. Although we did not directly quantify non-enzymatic antioxidants in the current experiment, our transcriptomic data implied potential elevation of glutathione-related pathways upon Si supply. Salt stress alone triggered significant increases in malondialdehyde (MDA) and superoxide anion (O_2_^−^) levels, accompanied by compensatory elevations in SOD, POD, and CAT activities that proved insufficient to fully offset oxidative damage [46,47]. Upon Si application, these enzymatic activities were further enhanced, while non-enzymatic antioxidants (glutathione and ascorbate) were also appreciably elevated, collectively driving a marked reduction in ROS accumulation and membrane lipid peroxidation. This coordinated upregulation of both enzymatic and non-enzymatic antioxidant systems mirrors findings in tomato and other crops [48,49], reinforcing the notion that Si acts as a broad-spectrum enhancer of plant oxidative defense. Importantly, the simultaneous enhancement of multiple antioxidant components suggests that Si orchestrates a systemic redox buffering network rather than simply activating a single detoxification route. Such a multi-layered strategy is conceptually advantageous for coping with the complex nature of salt-induced oxidative challenge, where different ROS species are generated in various subcellular compartments and require coordinated scavenging activities operating in parallel.

Consistent with our physiological observations, our transcriptome data demonstrated that Si remodels salt-triggered transcriptional reprogramming in potato roots and shoots. We found that Si markedly reduced the number of salt-responsive DEGs, especially in roots, where down-regulated transcripts predominated after Si treatment. This transcriptional quenching effect supports the view of Si as a ‘homeostatic buffer’ that attenuates excessive stress signaling rather than merely superimposing novel pathways [50]. From a mechanistic perspective, this observation is particularly telling: instead of functioning as a potent transcriptional activator that forces massive gene expression reprogramming, Si appears to normalize the stress-induced transcriptome toward a more ‘ground-state’ configuration. This mode of action may be metabolically more economical for the plant, as it avoids the fitness costs associated with wholesale transcriptional remodeling and allows resources to be channeled toward maintaining core physiological functions.

Our KEGG enrichment results uncovered clear tissue-specific transcriptional reprogramming triggered by Si under salt stress. In roots, Si suppressed enrichment of upstream MAPK and hormone-signaling DEGs while enriching peroxisomal ROS-scavenging, proline metabolism, and ABC-transporter-related pathways. In stem tissues, Si preferentially enhanced secondary-metabolite biosynthesis pathways including phenylpropanoid, flavonoid, and cutin–suberin synthesis. In roots, Si shifted the metabolic prioritization from upstream MAPK cascades and hormone perception toward peroxisomal ROS scavenging, fatty acid β-oxidation, proline accumulation, and ABC transporter-mediated ion compartmentalization, thereby enhancing energy recovery and nitrogen recycling. This reorientation toward peroxisomal and metabolic homeostasis in roots is biologically plausible, as roots are the first organs to perceive soil salinity and bear the primary burden of ion exclusion. By dampening upstream signal amplification and reinforcing downstream execution pathways, Si appears to streamline the root’s stress response into a more efficient mode. In leaves, Si directly boosted secondary metabolic pathways—including cutin/suberin, phenylpropanoid, flavonoid, and carotenoid biosynthesis—to synergistically reinforce physical cell wall barriers and non-enzymatic antioxidant capacity [51]. This leaf-specific strategy aligns with the physiological role of shoots as photosynthetic organs: rather than coping with direct ion toxicity, leaves face salt-induced water deficit and photo-oxidative stress, where cuticular waxes, phenylpropanoids, and carotenoids provide both physical protection and chemical quenching of ROS. The divergence between root and leaf transcriptional responses underscores that Si tailors its protective responses to the specific physiological demands of each organ.

To link transcriptome variation to our measured physiological traits, we performed weighted gene co-expression network analysis (WGCNA). It should be emphasized that WGCNA identifies gene modules and hub genes showing statistical correlation with phenotypic traits; this analysis only establishes associative relationships and cannot confirm direct causal or regulatory interactions between hub genes and salt-stress tolerance [52,53]. Accordingly, all hub genes identified here represent promising candidate genes for Si-mediated salt responses, and their biological functions need further experimental validation. We identified four salt-responsive modules (MElightgreen, MEblue, MEroyalblue, and MEturquoise) with distinct phenotypic associations and functional enrichments. MElightgreen, enriched in translational regulation and basal metabolism genes, showed significant positive correlations with CAT, POD, SOD, and proline, positioning it as a candidate core module potentially participating in enzymatic antioxidant defense and osmotic adjustment in aboveground tissues. MEblue, harboring RNA processing and sugar metabolism factors (CORK1, SUS1), exerted protection independently of the enzymatic antioxidant system—primarily through maintaining water balance and membrane stability—consistent with root-preferred strategies that prioritize ion and energy homeostasis, classifying it as a candidate ‘energy-saving’ homeostatic module [54]. MEturquoise encompassed glutathione synthesis, ABC transporters, and dehydrins, emphasizing secondary metabolic antioxidant defense and membrane transport [55,56], whereas MEroyalblue, containing hypoxia-induced protein (Hb1) and LEA proteins, responded specifically to severe membrane damage and water loss, and may serve as a potential stress-injury-related marker module [57]. The modular architecture reveals that antioxidant capacity is not a monolithic trait but is encoded by functionally specialized, independently regulated gene sets. The identification of MEturquoise as a glutathione-centric module is particularly relevant to the scope of this journal, as glutathione is not only a major cellular redox buffer but also a regulator of protein thiol redox status. Collectively, these modules function in a tissue-coordinated manner, and their hub genes are statistically associated with three interconnected physiological hubs: (1) protein homeostasis and translational regulation, (2) osmotic and carbon metabolic adjustment, and (3) ROS scavenging and redox homeostasis; however, direct regulatory links remain unproven in potato.

From these correlated module networks, we selected eight hub candidate genes for qPCR expression validation. Our qPCR results demonstrated that the expression trends of these eight genes were consistent with RNA-seq FPKM data, confirming the reliability of transcriptome expression profiles. Nevertheless, consistent expression patterns do not prove causal roles in Si-driven salt tolerance. *StALY1* (RNA-binding, regulating stress-responsive mRNA nuclear export), *StRPL6* (ribosomal protein sustaining translation under salinity), *StEF1A* (elongation factor orchestrating stress-induced protein synthesis), and *StBT1* (BTB-TAZ domain protein mediating ABA-dependent protein turnover via 26S proteasome) collectively safeguard protein homeostasis under salt stress. The inclusion of four distinct proteostasis-related genes underscores the critical importance of maintaining a functional translational machinery under salt stress—a point often overshadowed by the traditional focus on antioxidant enzymes. Salt stress causes widespread protein misfolding, and the coordinated action of RNA-binding proteins, ribosomal subunits, elongation factors, and ubiquitin–proteasome components suggests that Si actively preserves proteome quality and quantity. *StSUS1* (sucrose synthase) and *StLEA* (late embryogenesis abundant protein) coordinate osmotic adjustment and carbon metabolic homeostasis. LEA proteins act as chaperone-like protectors of membrane integrity under dehydration, while sucrose synthase fuels osmotic adjustment through soluble sugar accumulation and provides carbon skeletons for secondary metabolism. *StPRX52* (peroxidase involved in cell-wall remodeling and ROS scavenging) and *StGSH1* (glutathione synthesis rate-limiting enzyme) reinforce the antioxidative defense system. The identification of *StGSH1* as a hub gene is particularly significant because it directly connects Si action to the cellular redox buffer capacity. Similarly, *StPRX52* not only scavenges H_2_O_2_ but also participates in lignin and suberin deposition, bridging ROS detoxification with cell-wall strengthening. We speculate that these three pathways (proteostasis, osmotic balance, ROS elimination) may synergistically contribute to Si-improved salt tolerance in potato; this hypothesis requires further genetic evidence from gene knockout or overexpression assays. Importantly, these pathways are intimately interconnected: maintenance of protein homeostasis ensures sustained synthesis of antioxidant enzymes and osmolyte biosynthetic enzymes, while glutathione modulates protein redox status through glutathionylation, influencing numerous signaling and metabolic proteins.

Several limitations should be acknowledged. First, qPCR validation was confined to eight core genes; broader validation would enhance robustness. Second, although we have identified candidate hub genes and associated pathways, causal relationships await functional validation through knockout or overexpression studies. Third, in vitro conditions cannot fully recapitulate field environments; field trials with soil-applied Si are necessary to translate these findings into agricultural practice. Fourth, we did not quantify tissue silicon accumulation in the present study. Measuring Si concentrations in potato tissues will help establish direct links between exogenous Si supply, tissue Si loading, and the observed salt-alleviating phenotypes in future work. From a mechanistic standpoint, the cascade interactions among protein homeostasis, osmotic regulation, and ROS redox networks warrant further exploration via spatiotemporal expression profiling, protein–protein interaction assays, and cytological approaches. The specific regulatory hierarchies upstream and downstream of *StALY1*, *StRPL6*, *StEF1A*, *StBT1* (proteostasis), *StSUS1*, *StLEA* (osmotic adjustment), *StGSH1*, and *StPRX52* (antioxidant metabolism) offer promising research directions for subsequent gene functional characterization and molecular breeding for salt tolerance.

## 5. Conclusions

**Figure 10:**
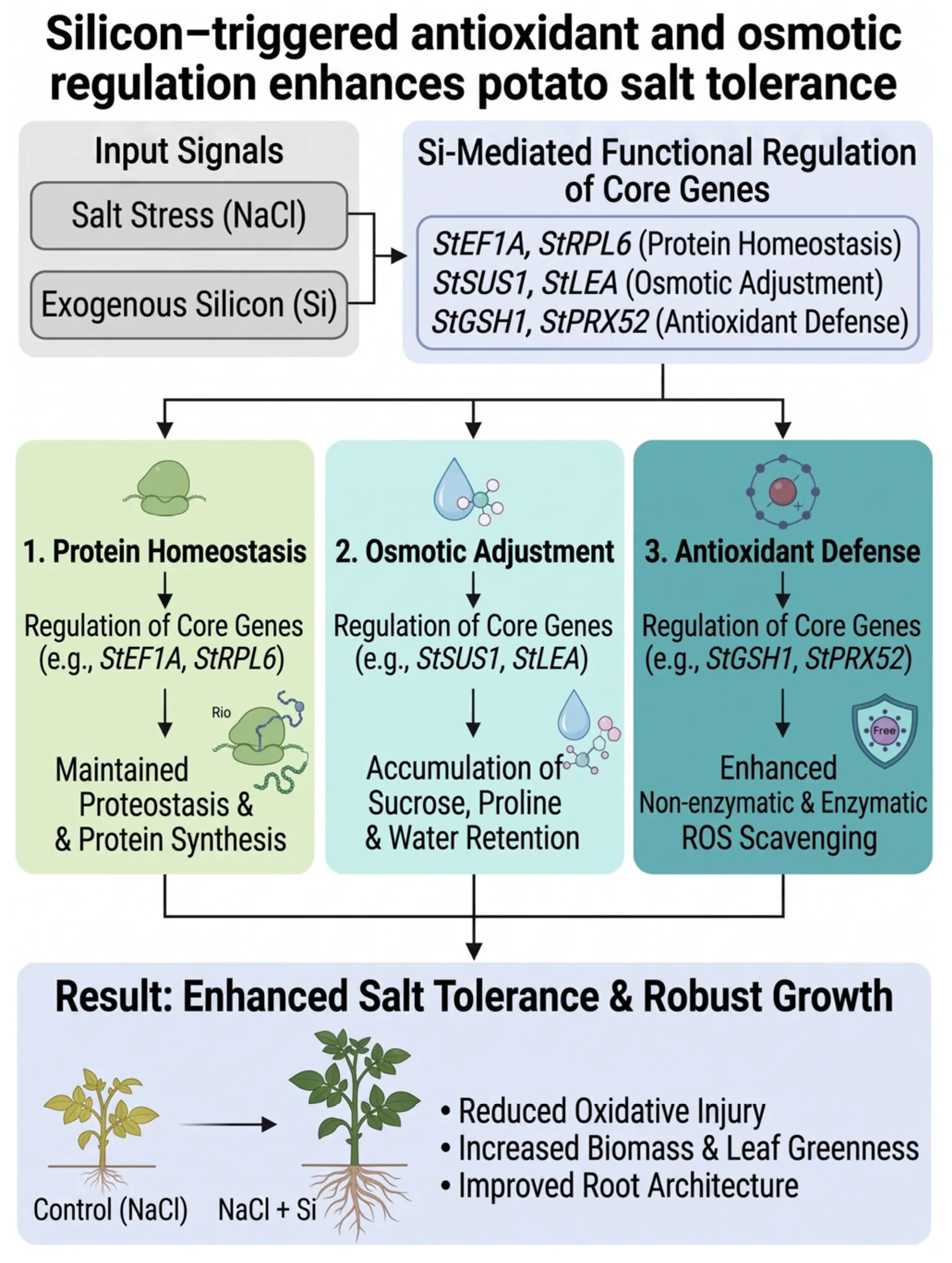
Schematic model for silicon-mediated salt-tolerance regulation in potato.

In summary, exogenous silicon improved potato salt tolerance in a concentration-dependent manner. The optimal Si concentrations were 1.0 mM for tissue-cultured seedlings and 2.0 mM for potted plants. Moderate silicon relieved salt-induced ROS burst and oxidative damage by enhancing antioxidant enzyme activities, stabilizing redox homeostasis and osmotic balance. Transcriptome-based WGCNA and RT-qPCR validation identified eight hub-genes involved in protein homeostasis, osmotic adjustment, and ROS-scavenging antioxidant defense, which underpin silicon-mediated salt responses (Figure 10). Our results demonstrate that boosted antioxidant capacity acts as a core mechanism of Si-conferred salt tolerance. This study provides optimized silicon application strategies and valuable candidate genes for potato salt-tolerance research.

## Data Availability

The original contributions presented in this study are included in the article/Supplementary Material. Further inquiries can be directed to the corresponding authors.

## Supplementary Materials

The following supporting information can be downloaded at: https://www.mdpi.com/article/10.3390/antiox15101247/s1, Table S1: Gene IDs of the four candidate WGCNA modules used in co-expression network visualization and hub-gene identification; Table S2: Functional annotation of the core genes of the Melightgreen module; Table S3: Functional annotation of the core genes of the Meblue module; Table S4: Functional annotation of the core genes of the Meroyalblue module; Table S5: Functional annotation of the core genes of the Meturquoise module. Figure S1: Volcano plots for transcriptomic differential-expression analysis; Figure S2: Weighted gene co-expression network analysis (WGCNA).
