# Supplementary material for: Silicon Confers Salt Tolerance in Potato in a Concentration-Dependent Manner via Coordinated Antioxidant and Osmotic Regulation
Source: Antioxidants (Basel). 2026 Sep 27;15(10):1247. doi: 10.3390/antiox15101247 (PMC13700833; doi:10.3390/antiox15101247)
Supplement: Supplementary file 1 [file antioxidants-15-01247-s001.zip › Supplementary information.pdf]

Supplementary information

Silicon confers salt tolerance in potato in a concentration-dependent manner via coordinated antioxidant and osmotic regulation

Panfeng Yao [1,2], Junmei Cui [1], Wenlin Li [3], Ying Wang [1], Jianguo Wei [1], Zhenzhen Bi [1,2], Chao Sun [1,2], Zhen Liu [1,2], Han Wang [2], Zhanshu Song [4], Yuhui Liu [1,2,*], Jiangping Bai [5,*]

[1] State Key Laboratory of Aridland Crop Science, Gansu Agricultural University, Lanzhou 730070, China; yaopf@gsau.edu.cn (P.Y.);

[2] College of Agronomy/Seed Industry Research Institute of Gansu Provincial University, Gansu Agricultural University, Lanzhou 730070, China

[3] College of Horticulture, Gansu Agricultural University, Lanzhou 730070, China

[4] Qingyang City Academy of Agricultural Sciences, Qingyang 74500, China

[5] Lanzhou Institute of Technology, Lanzhou 730070, China

* Correspondence: lyhui@gsau.edu.cn (Y.L.); baijp@lzit.edu.cn (J.B)

Table S2. Functional annotation of the core genes of the Melightgreen module.

Module	Hub gene	Hub gene in A.thaliana	Gene fuction	Gene nomenclature
Melightgreen	Soltu.DM.11G022490	AT5G60390	Alpha subunit of the elongation factor-1 complex.	EF1A
Melightgreen	Soltu.DM.03G033060	AT1G48570	Zinc finger (Ran-binding) family protein.	ZFR1
Melightgreen	Soltu.DM.05G022170	AT5G47030	Mitochondrial ATP synthase subunit delta.	ATPδ1
Melightgreen	Soltu.DM.09G008720	AT5G01310	Adenylylsulfate sulfohydrolase activity in vitro.	APTX1
Melightgreen	Soltu.DM.01G039390	AT3G05500	Lipid droplet surfaces and shares sequence homology with family of small rubber particle proteins.	LDAP3
Melightgreen	Soltu.DM.04G033650	AT5G42900	Key regulatory factor in the COP1-HY5 regulatory hub.	OR27
Melightgreen	Soltu.DM.05G000800	AT4G24830	Arginosuccinate synthase family.	ASS1

Table S3. Functional annotation of the core genes of the Meblue module.

Module	Hub gene	Hub gene in A.thaliana	Gene fuction	Gene nomenclature
Meblue	Soltu.DM.04G027280	-	Alba DNA/RNA-binding protein.	ALBA1
Meblue	Soltu.DM.06G033070	AT2G26970	Polynucleotidyl transferase, ribonuclease H-like superfamily.	PNT1
Meblue	Soltu.DM.10G020650	AT5G59950	RNA-binding (RRM/RBD/RNP motifs) family protein.	ALY1
Meblue	Soltu.DM.04G009150	AT1G74050	Ribosomal protein L6 family protein;	RPL6
Meblue	Soltu.DM.01G039460	AT5G27730	heparan-alpha-glucosaminide N-acetyltransferase-like protein	HGSNAT1
Meblue	Soltu.DM.11G001150	AT1G56145	LRR-malectin receptor kinase; required for cellooligomer-induced responses.	CORK1
Meblue	Soltu.DM.03G025100	AT5G63160	BTB and TAZ domain protein.	BT1
Meblue	Soltu.DM.07G013360	AT5G20830	Encodes a protein with sucrose synthase activity (SUS1).	SUS1
Meblue	Soltu.DM.04G027280	-	Alba DNA/RNA-binding protein.	ALBA1
Meblue	Soltu.DM.06G033070	AT2G26970	Polynucleotidyl transferase, ribonuclease H-like superfamily.	PNT1
Meblue	Soltu.DM.10G020650	AT5G59950	RNA-binding (RRM/RBD/RNP motifs) family protein.	ALY1

Table S4. Functional annotation of the core genes of the Meroyalblue module.

Module	Hub gene	Hub gene in A.thaliana	Gene fuction	Gene nomenclature
Meroyalblue	Soltu.DM.07G003670	AT2G16060	Class 1 nonsymbiotic hemoglobin induced by low oxygen levels with very high oxygen affinity.	Hb1
Meroyalblue	Soltu.DM.06G032730	AT5G05340	Sequence similarity to peroxidases that is involved in lignin biosynthesis.	PRX52
Meroyalblue	Soltu.DM.05G024800	AT4G02340	alpha/beta-Hydrolases superfamily protein.	EH1
Meroyalblue	Soltu.DM.03G030260	AT1G18810	phytochrome kinase substrate-like protein.	PKS3
Meroyalblue	Soltu.DM.07G024790	AT5G54370	Late embryogenesis abundant (LEA) protein-like protein.	LEA

Table S5. Functional annotation of the core genes of the Meturquoise module.

Module	Hub gene	Hub gene in A.thaliana	Gene fuction	Gene nomenclature
Meturquoise	Soltu.DM.02G019450	AT3G25830	Encodes the monoterpene 1,8-cineole synthase, atTPS-Cin.	TPS
Meturquoise	Soltu.DM.11G003810	AT1G65610	Six-hairpin glycosidases superfamily protein.	KOR2

Meturquoise	Soltu.DM.02G006200	-	Dehydrin domain containing protein.	DHN
Meturquoise	Soltu.DM.09G023380	AT2G47800	Plasma membrane localized ATPase transporter involved in multidrug transport.	MRP4
Meturquoise	Soltu.DM.08G018020	AT2G39030	Acts as an ornithine N-delta-acetyltransferase, leading to the formation of N-delta-actetylornithine.	NATA1
Meturquoise	Soltu.DM.08G027770	AT4G23100	Glutamate-cysteine ligase catalyzing the first, and rate-limiting, step of glutathione biosynthesis.	GSH1
Meturquoise	Soltu.DM.03G026010	AT3G26600	Armadillo repeat protein.	ARO4

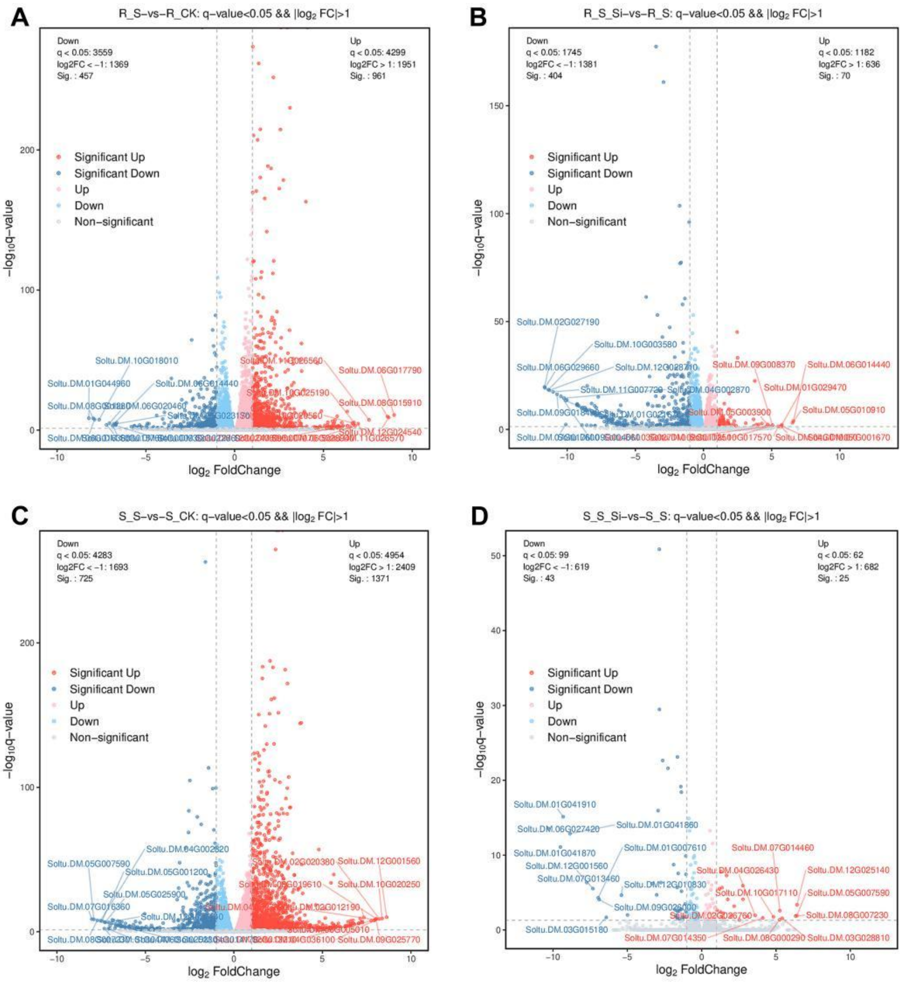

Figure S1. Volcano plots for transcriptomic differential-expression analysis. (A) Root: R-CK vs R-S; (B) Root: R-S vs R-S-Si; (C) Stem: S-CK vs S-S; (D) Stem: S-S vs S-S-Si. The x-axis shows $\log_2$ fold-change of gene expression, and the y-axis denotes $-\log_{10}$ *p*-value for differential significance. Red dots: significantly up-regulated genes; blue dots: significantly down-regulated genes; gray dots: non-significant genes.

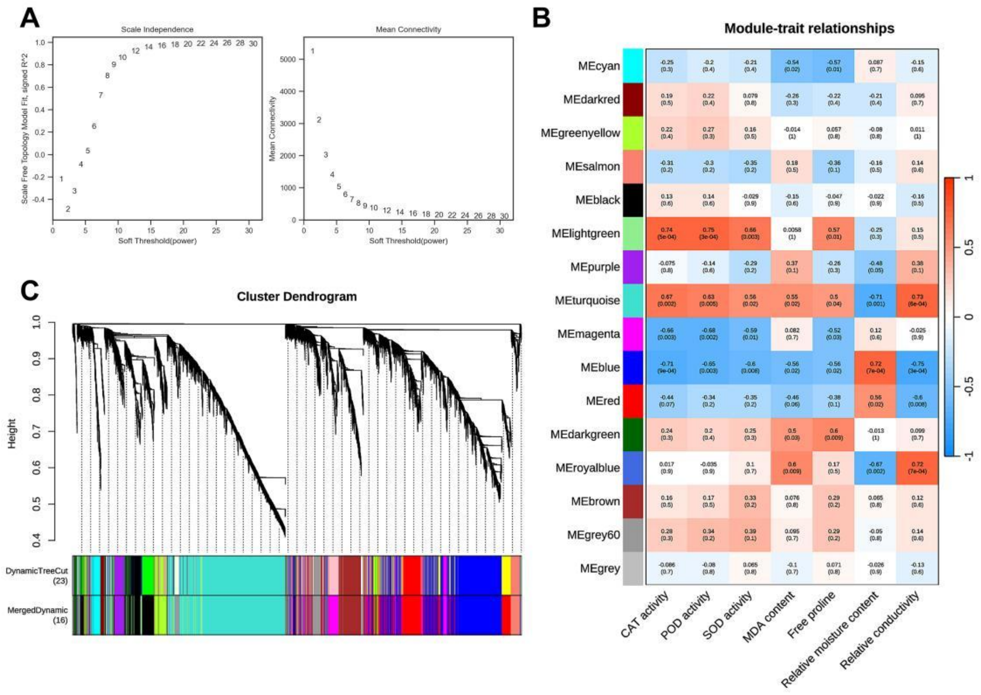

Figure S2. Weighted gene co-expression network analysis (WGCNA). (A) Determination of soft-thresholding power for scale-free network construction. Left: scale-free fit index; right: mean connectivity. (B) Heatmap of module-trait correlations. Colours represent Pearson correlation coefficients, with corresponding p-values in parentheses. (C) Gene hierarchical clustering dendrogram. Lower colour bars show modules assigned by DynamicTreeCut and after module merging.
